# Memory-Based Pruning of Deep Neural Networks for IoT Devices Applied to Flood Detection

**DOI:** 10.3390/s21227506

**Published:** 2021-11-12

**Authors:** Francisco Erivaldo Fernandes Junior, Luis Gustavo Nonato, Caetano Mazzoni Ranieri, Jó Ueyama

**Affiliations:** 1 SIDIA R&D Institute, Manaus 69055-035, Brazil; 2 Institute of Mathematical and Computer Sciences, University of São Paulo (USP), São Carlos 13566-590, Brazil; gnonato@icmc.usp.br (L.G.N.); cmranieri@alumni.usp.br (C.M.R.); joueyama@icmc.usp.br (J.U.)

**Keywords:** deep neural networks, semantic segmentation, random pruning, Internet of Things, flood detection, user preference

## Abstract

Automatic flood detection may be an important component for triggering damage control systems and minimizing the risk of social or economic impacts caused by flooding. Riverside images from regular cameras are a widely available resource that can be used for tackling this problem. Nevertheless, state-of-the-art neural networks, the most suitable approach for this type of computer vision task, are usually resource-consuming, which poses a challenge for deploying these models within low-capability Internet of Things (IoT) devices with unstable internet connections. In this work, we propose a deep neural network (DNN) architecture pruning algorithm capable of finding a pruned version of a given DNN within a user-specified memory footprint. Our results demonstrate that our proposed algorithm can find a pruned DNN model with the specified memory footprint with little to no degradation of its segmentation performance. Finally, we show that our algorithm can be used in a memory-constraint wireless sensor network (WSN) employed to detect flooding events of urban rivers, and the resulting pruned models have competitive results compared with the original models.

## 1. Introduction

Flood risk is the probability that exposure to flooding will cause negative consequences, ranging from economic losses to social and health issues [1]. When this risk is not negligible, flood management solutions become of paramount importance, and different types of technologies have been proposed for this aim [2]. Within this context, automatic flood detection may be important to trigger alerts or damage control systems.

A method commonly applied to measure the water level of a river is the implantation of gauges at different locations of its course [3,4,5]. Although well-established and suitable for most situations, this solution is unable to detect uncommon events, such as extreme flooding. Other approaches rely on satellite or airborne images [6,7], which can provide good predictions when fed to state-of-the-art machine learning techniques. However, data from these sources are not suitable for real-time predictions, since it depends on the overpasses of satellites or other devices, which sometimes happens only as often as once or twice a day.

An alternative is the placement of still cameras at the basin of a river [8]. Although each particular camera has limited coverage, which means that multiple cameras at different locations may be needed to cover a broader area, this approach has low cost of implementation and does not require a sophisticated infrastructure for deployment: a set of low-end Internet of Things (IoT) devices may be sufficient for data gathering. In Vandaele et al. [8], opportunistic data from a network of cameras placed throughout the courses of two rivers were employed to detect flooding based on deep learning approaches for segmentation. Our work is developed within this same context, in which a wireless sensor network (WSN) was employed to provide images from still cameras at the basins of the rivers of the city of São Carlos, Brazil [9,10,11,12,13] and upload them to a cloud server for flood prediction.

Although deep neural networks (DNN), particularly convolutional neural networks (CNN), may provide predictions with high accuracy for image data in general, the resulting models are usually large, with millions of parameters [14]. Therefore, there are requirements for computational resources to store the models and process the inferences, which may not be met by IoT devices such as those within our WSN. In this scenario, the most straightforward solution is to upload the images to a cloud server, which may perform predictions as a microservice [15]. However, since many of the river cameras may be placed in remote locations with an unstable internet connection, the uploads of data to the cloud server may often fail, increasing the risk of not triggering damage control protocols during important flood events.

A possible solution is to not only deploy the flood detection software in the cloud server, but to also deploy a lighter version at the edge (i.e., within the nodes of the WSN), which is challenging because the sensors themselves usually do not have enough memory to run state-of-the-art DNN models. The present work addresses this issue by proposing a DNN pruning algorithm [16] based on a randomised heuristic. The proposed pruning algorithm can find a suitable pruned DNN model within a specified amount of memory available in the target device by randomly removing convolutional filters in a model.

We applied the proposed algorithm to prune a semantic segmentation DNN model and used this model in our WSN to perform flood detection from river images of still cameras. It is important to notice that the pruning procedure is performed in a powerful workstation, and once the pruned DNN model is found, it can be deployed on an IoT device. The proposed algorithm allows an individual sensor to perform semantic segmentation even if it cannot connect to its central server. It consists of iteratively eliminating convolutional filters until the model reaches the specified amount of memory. Moreover, to speed up the pruning procedure, once a convolutional filter has been removed from the DNN model, it cannot be added back, a heuristic that reduces the searching space of possible combinations of convolutional filters.

The algorithm was employed for pruning the DeepLabv3 DNN models [17], considered the best semantic segmentation models currently available, and the results show that this strategy can find pruned models within the specified amount of memory with good segmentation performance. Besides performing experiments with the dataset of riverside images from Sao Carlos, the present work also consists of an additional set of experiments using a public dataset, specifically, the PennFundan dataset, for pedestrian detection. This was done as an additional test for the performance of the model in tasks other than river segmentation. Results have shown that expressive reductions in the memory footprint of the model were achieved at minimal cost regarding the Intersection over Union (IoU) in the test set for both datasets.

Finally, the contributions of this work can be summarised as follows:

- We propose a heuristic that allows the reduction of the memory footprint of DNNs applied in semantic segmentation tasks;
- We propose a pruning algorithm that finds DNN models with a user-specified amount of memory, allowing their deployment on memory-constrained devices, such as IoT and edge devices;
- We propose using DNNs in an IoT-based river flooding detection system, which allows a city’s authorities and population to monitor and act during dangerous thunderstorms.

The remainder of this work gives more details about the proposed DNN memory-based pruning algorithm, and it is organised as follows. The background materials related to DNN semantic segmentation and pruning are presented in Section 2. The proposed pruning algorithm is defined in Section 3. We describe the methodology devised to validate our algorithm in Section 4. Next, the results obtained are presented in Section 5. Finally, the conclusion and suggestions for future work are detailed in Section 6.

## 2. Background

This section presents the theoretical background used in our proposed algorithm. It first explains semantic segmentation through deep neural network (DNN) models. Then, it shows how we are using semantic segmentation to detect the level of urban rivers. Lastly, it shows how to reduce the computational complexity of DNN models with convolutional filter pruning.

### 2.1. DNN for Semantic Segmentation

Semantic segmentation is a computer vision technique where each pixel of a given single image is assigned to one class or label [18]. In other words, semantic segmentation allows us to know the location of objects and people in images. Its main difference from instance segmentation is that it cannot locate individual instances of a specific object [18]. For example, if there are two mugs in a given image, semantic segmentation could be used to find the mugs’ location in the image, but it could not find the number of mugs. Furthermore, semantic segmentation tries to find the exact contour or boundaries of an object in an image, while a similar technique called object detection only tries to find the center location of the objects and put a rectangular box around them [18].

**Figure 1:**
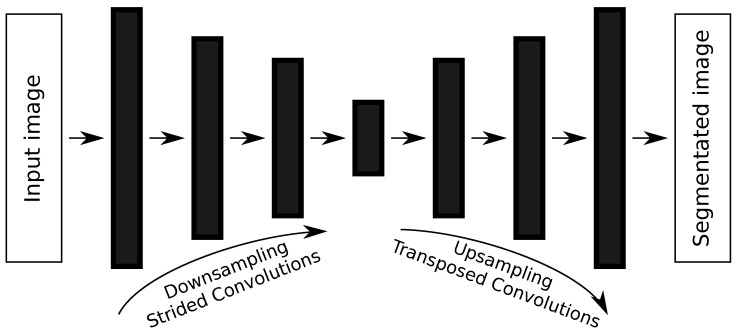
Example of a traditional DNN topology used for semantic segmentation.

Recently, DNN models have become powerful enough to perform semantic segmentation. One of the most popular DNN topologies used in semantic segmentation resembles an autoencoder [19]. In this sense, the first half of the DNN model is responsible for downsampling the input image until it reaches a low dimensional latent representation. In contrast, the other half performs the segmentation by upsampling this latent representation to the original input image’s size. The downsampling operations are generally performed using *strided convolutions*, while the upsampling operations are performed by *transposed convolutions* [20], as illustrated in Figure 1. For example, for medical imaging diagnostics, Ronneberger et al. [21] developed the U-Net to segment blood vessels and organs from a diverse set of images. Likewise, the *deconvolution network* developed by Noh et al. [22] is another example of a DNN used for segmentation, but this model is used with common daily images instead of medical ones.

**Figure 2:**
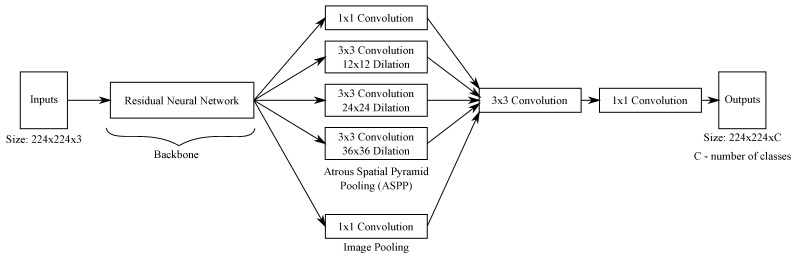
DeepLabv3 neural network used for semantic segmentation.

Currently, semantic segmentation is so popular that even Microsoft has developed the Common Object in Context (COCO) dataset [23], used as a benchmarking tool by many researchers in the field. The DeepLabv3 DNN models developed by Chen et al. [17] are among the state-of-the-art models in the COCO dataset. These models use residual neural networks (ResNets) [24] in conjunction with atrous spatial pyramid pooling (ASPP), which consists of a set of convolution operations with different dilation sizes, as illustrated in Figure 2.

### 2.2. Semantic Segmentation for River Level Detection

Because of its powerful segmentation capabilities, we are currently using the DeepLabv3 DNN model to aid in our task of detecting the water level of urban rivers. The DeepLabv3 is used to segment the water surface from a given image, which is then used to compute the water level. We follow four steps to detect a river’s water level:

1. Video cameras installed at the riverbed collect images of the monitored rivers;
2. The DeepLabv3 DNN model is used to segment and identify the water surface in the collected image;
3. Using a manually drawn reference line perpendicular to the water surface, we compute the river water level;
4. If the water level in any monitored rivers reaches a manually set threshold, flooding alerts are sent to the population.

**Figure 3:**
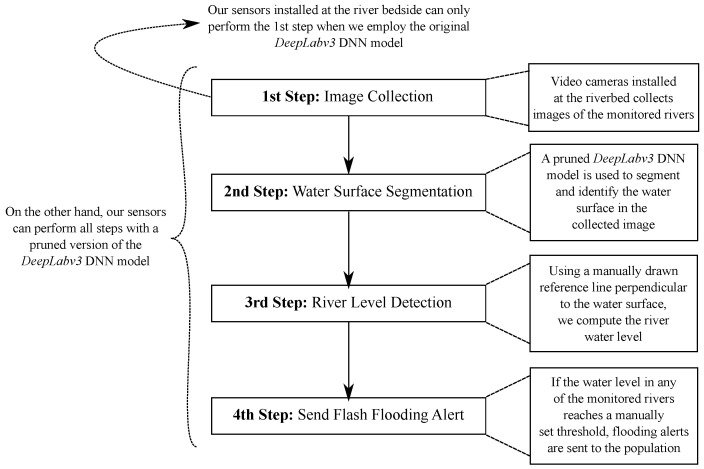
Overview of the proposed river flooding detection system.

**Figure 4:**
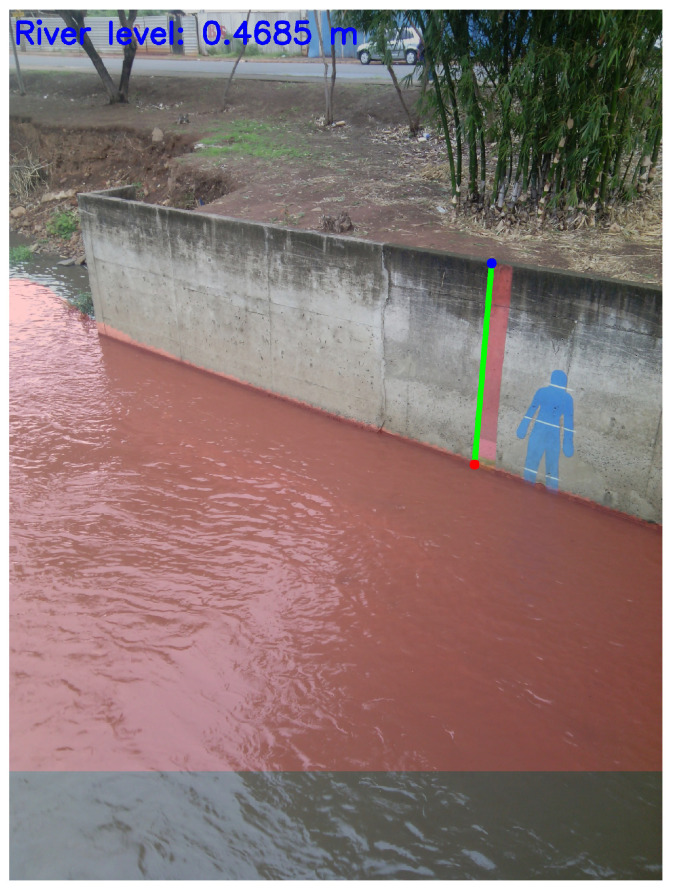
River level detection aided by the DeepLabv3 DNN model.

Figure 3 illustrates the aforementioned procedure. The sensor network installed at the river bedside can only perform all four steps when we employ a pruned version of the DeepLabv3 DNN model. One example of how a river’s water level segmented (red surface) by a DeepLabv3 model is shown in Figure 4. The green line is the reference line, and the *Euclidean distance* between the red and blue dots in Figure 4 is used to compute the river level.

Currently, because our sensors installed at the riverbed side do not have enough computational power to run a DNN model, all collected images are sent through the internet to a cloud server running a DeepLabv3 model. Our sensors have a Raspberry Pi 3 Model B with only 1 GB of RAM available, which is not enough to run most DNN models. Thus, the proposed work aims to enable the sensors to perform segmentations locally independently of the internet’s availability.

### 2.3. Convolutional Filter Pruning

*Convolutional filter pruning* is a popular technique used to reduce the computational and memory complexity of DNNs. Each convolutional layer’s parameters are represented by a four-dimensional tensor of size given by O×D×W×H, where *O* is the number of filters, while *D*, *W*, and *H* is the depth, width, and height, respectively, of each one of the *O* filters. In this case, to reduce this layer’s computational complexity, one should eliminate a *K* number of filters, 1≤K≤O−1. This procedure can be performed with any type of convolutional layer from strided convolutions to transposed convolution operations. The methodologies in [16,25,26,27] relies on filter removal to prune a DNN, but they used different selection criteria to choose which filters to keep and which filters to eliminate. These selection criteria range from the $l_{0}$ norm to the entropy of each filter. However, some works show that randomized heuristics performs as well as pre-defined selection criteria [28,29,30]. Thus, our proposed *DNN memory-based pruning* algorithm eliminates convolutional filters at random, but it is controlled by a specified amount of memory to deploy the model in a memory constraint device. The following section gives more details about our algorithm.

## 3. Proposed DNN Memory-Based Pruning Algorithm

We developed a DNN architecture pruning algorithm in which the memory footprint of a given DNN model is reduced by randomly eliminating convolutional filters. Binary numbers represent each filter, where a ‘1’ is used for filters that will *not* be eliminated and a ‘0’ for the ones that will be. Moreover, the pruning process is controlled by a user’s chosen memory footprint. The overall pruning algorithm is presented in Algorithm 1, and it works by finding the best candidate from a set of *N* candidate solutions, all of them with the specified memory footprint. Moreover, after the pruning procedure, each candidate solution is evaluated on the input dataset to verify its segmentation performance. This evaluation consists of a single epoch training session over the entire dataset to speed up the algorithm. In our case, the best candidate solution is the one with the highest mean Intersection-over-Union (IoU) [31] over all classes. The best candidate solution is then retrained in the chosen dataset, with a *fine-tuning* process, to recover any segmentation performance loss caused by the pruning procedure.
**Algorithm 1.** Overview of the pruning algorithm **Input** :**Pruning parameters:** Number of candidate solutions (*N*), desired      memory footprint (mem), probability of removing a filter (*p*), learning      rate for memory evaluation ($\alpha_{mem}$). **Training parameters:** input dataset      (X), number of epochs for fine-tuning (*e*), learning rate for fine-tuning (α),      learning rate decay schedule step (*s*), learning rate decay (γ). **Shared**
     **parameters:** original DNN model (DNN). **Output**: Pruned DNN model with the desired memory footprint (prunedDNN).

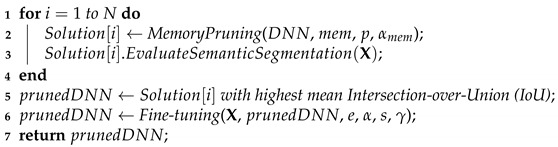


**Figure 5:**
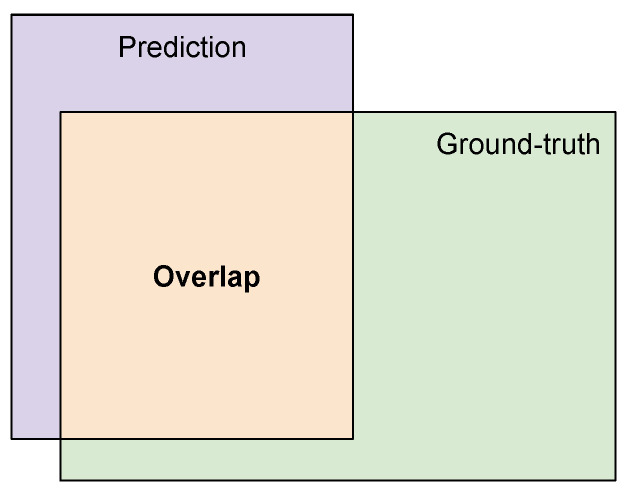
Intersection-over-Union (IoU) results from the ratio between the area of overlap between the predicted mask and the ground truth (intersection), and the total area occupied by them (union).

The Intersection-over-Union (IoU) is the most popular evaluation criteria for semantic segmentation and object localization tasks [31]. It consists of the ratio between the intersection and the union areas between the annotated mask (i.e., ground truth) and the mask segmented using the machine learning model (i.e., prediction). Because the intersection is actually a subset of the union, the IoU might be a number in the [0,1] interval. An IoU of 1 means perfect overlap between the ground truth and the output of the segmentation model, while an IoU of zero means no overlap at all. More formally, the IoU can be defined as:

(1) $$IoU=\frac{|Y\cap \hat{Y}|}{|Y\cup \hat{Y}|}=\frac{Intersection}{Union},$$

where *Y* represents the ground-truth segmentation and $\hat{Y}$ is the predicted segmentation from the DNN model. Figure 5 illustrates this metric.

The algorithm receives as inputs the following list of parameters:

- The chosen DNN model to be pruned (DNN);
- Four parameters related to the pruning process:
  - The number of candidate solutions (*N*) that the algorithm will generate;
  The desired memory footprint, in bytes, of all solutions (mem);
  The probability of randomly removing a single convolutional filter ($p_{m}$);
  The learning rate used when evaluating the memory footprint of a candidate solution ($\alpha_{mem}$);
- Five parameters related to the fine-tuning procedure:
  - The chosen dataset used to train the pruned DNN model (X);
  The total number of epochs to retrain pruned DNN model (*e*);
  The initial learning rate (α);
  The number of epochs, or steps, the algorithm waits before reducing the learning rate (*s*);
  The learning rate decay factor (γ).

The pruning procedure itself is shown in Algorithm 2. It mainly consists of the elimination of convolutional filters until the candidate solution has reached the desired memory footprint. It is also important to note that the algorithm never adds back a filter that was eliminated. This strategy allows us to considerably reduce the searching space, speeding up the process of finding pruned DNN models. A single run of the proposed algorithm takes less than two hours to find a suitable pruned DNN model. Furthermore, the process of removing convolutional filters is applied to all types of convolutional layers, such as strided and transposed convolutions. The process of evaluating the memory footprint of a candidate solution (the method EvaluateMemoryFootprint) is performed with the help of the *Deep Learning API* we are employing. The memory is evaluated using the CPU model, because many IoT devices lack a GPU component.
**Algorithm 2.** DNN memory-based pruning (*MemoryPruning*) **Input** :Desired memory footprint (mem), probability of removing a filter (*p*),      learning rate for memory evaluation ($\alpha_{mem}$), original DNN model (DNN).
 **Output**: A pruned candidate solution with the desired memory footprint (Solution).
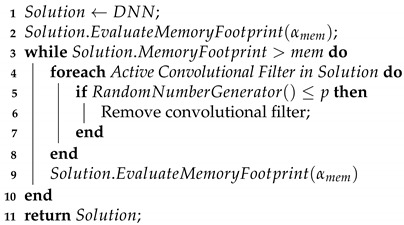


At the end of the whole process, a pruned DNN model (prunedDNN) is returned, and it can be deployed at the user’s chosen memory constraint device. We believe that our algorithm’s straightforwardness will allow it to be used by a range of researchers from different fields of studies. The results presented in the following sections demonstrate that this simple strategy is capable of finding pruned DNN models with competitive performance.

## 4. Experimental Design

The experimental design used to validate the proposed DNN memory-based pruning algorithm is presented in this section:

1. We discuss the DNN models in which we tested our algorithm;
2. We present the two semantic segmentation datasets that we chose to use;
3. We show the algorithm parameters used for our tests.

All results we present in this work were obtained using the PyTorch 1.7.1 DeepLearning API on Python 3.8 running on a Desktop PC equipped with an Nvidia RTX 3070 GPU, AMD Ryzen 7 3700X, and 32 GB of RAM.

### 4.1. Chosen DNN Architectures for Pruning

We have chosen to prune the DeepLabv3 DNN models because they are used in our IoT flood detection system. The DeepLabv3 models are composed of two main components, as illustrated in Figure 2: the backbone and the atrous spatial pyramid pooling (ASPP). In general, the backbone is composed of a residual neural network (ResNet) [24] responsible for most of the segmentation work, and it has a topology of downsampling and upsampling similar to Figure 1. On the other hand, the ASPP mainly comprises parallel dilated convolution operations, and it is responsible for improving the segmentation done by the backbone. However, the ASPP is only responsible for a small fraction of the memory footprint of a DeepLabv3 model. The ASPP contributes only to around 130 MB of the memory footprint. Hence, we are only pruning the backbone. Specifically, we are pruning the models with ResNets containing 50 layer (ResNet50) and 101 layer (ResNet101) backbones. Furthermore, we used DeepLabv3 models pre-trained on the Microsoft COCO 2017 dataset [23] and performed transfer learning [32] on our chosen datasets.

### 4.2. Chosen Semantic Segmentation Datasets

**Figure 6:**
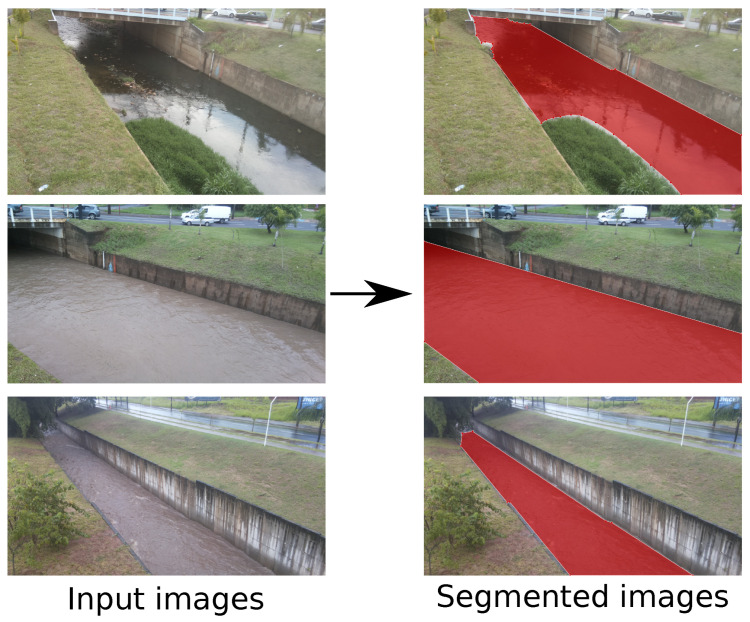
Sample images from our *São Carlos Rivers* dataset.

The DeepLabv3 DNN models previously mentioned were tested with two datasets: our custom dataset used for flood detection and the Penn–Fudan dataset for pedestrian detection. Our custom dataset, which we called *São Carlos Rivers*, is a collection of river images collected in São Carlos, SP, Brazil. Our dataset is comprised of 968 training images and 30 test images taken from two different rivers with different water levels, camera viewpoints, and lighting conditions. We manually annotated the water surface, or water blade, for each image. Moreover, all images of this dataset contain only two classes: water blade or background. Figure 6 shows some sample images from our custom dataset. The Penn–Fudan dataset was created by Wang et al. [33], and it has a total of 170 images of various pedestrians. We randomly divided the dataset into 136 images used for training and 34 used for testing. This dataset also contains only two classes: pedestrians and background.

We present the memory footprint and test IoU on both datasets of the original DeepLabv3 models in Table 1. The original DeepLabv3 model with a ResNet50 backbone has a memory footprint of $1.12\times 10^{9}$ bytes, while the model with a ResNet101 backbone has a footprint of $1.94\times 10^{9}$ bytes. The original ResNet50 backbone obtained a test IoU of 0.9404 and 0.6992 on the São Carlos Rivers and Penn–Fudan datasets, respectively. Similarly, the original ResNet101 backbone obtained a test IoU of 0.9412 and 0.6779 on the São Carlos Rivers and Penn–Fudan datasets. Table 1 presents the original models’ results without pruning; thus, we compare the following section results with those from Table 1.

### 4.3. Algorithm Parameters

All results presented in the following section were obtained using the parameters shown in Table 2. We have specified two amounts of memory footprint: $1.07\times 10^{9}$ and $5.37\times 10^{8}$ bytes. These memory footprints are around 1 GB and 512 MB of memory, which is more than enough to run the DeepLabv3 DNNs on our IoT devices. Moreover, to obtain statistically significant results, we used 30 candidate solutions for each run of the algorithm and a total of 30 independent runs of the proposed algorithm.

## 5. Experimental Results

This section details the obtained results using our proposed algorithm with our chosen DNN architectures, semantic segmentation datasets, and guidelines to help deploy the proposed algorithm on real hardware. It is important to note that the results are from a set of 30 independent runs.

### 5.1. Results

As we specified in the previous section, we evaluated the proposed algorithm on two different DeepLabv3 backbones with two different datasets using two different memory footprints: 1 GB and 512 MB footprints. Hence, there are a total of eight sets of results, as summarized in Table 3. To help with the results’ organization, we first present the ones for the chosen 1 GB memory footprint, followed by the 512 MB memory footprint.

**Figure 7:**
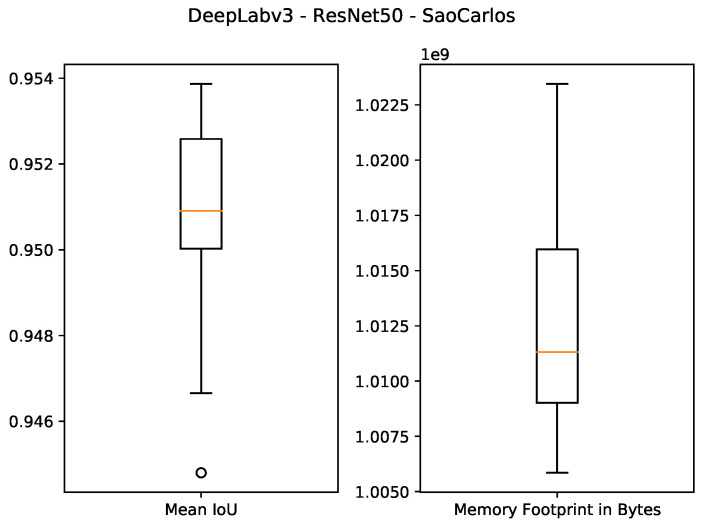
Boxplot results for the *São Carlos Rivers* dataset using the *ResNet50* backbone and 1 GB of desired memory footprint.

**Figure 8:**
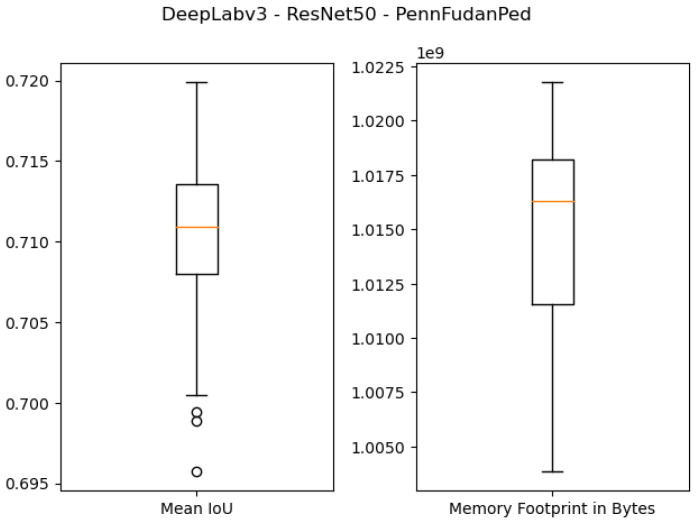
Boxplot results for the *Penn–Fudan* dataset using the *ResNet50* backbone and 1 GB of desired memory footprint.

**Figure 9:**
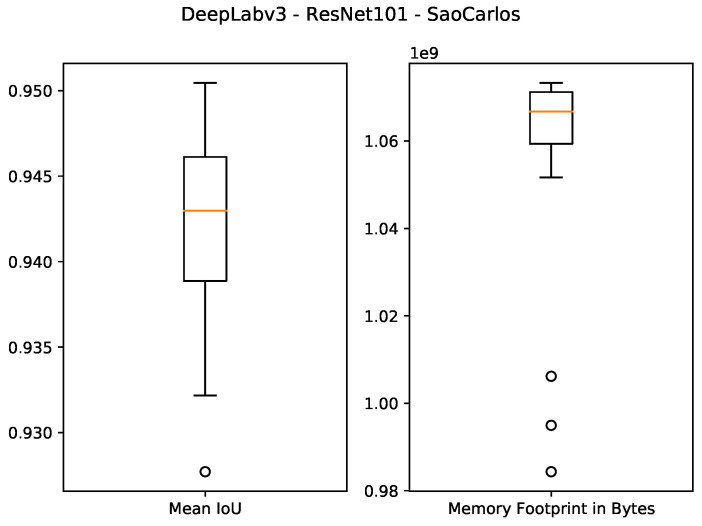
Boxplot results for the *São Carlos Rivers* dataset using the *ResNet101* backbone and 1 GB of desired memory footprint.

**Figure 10:**
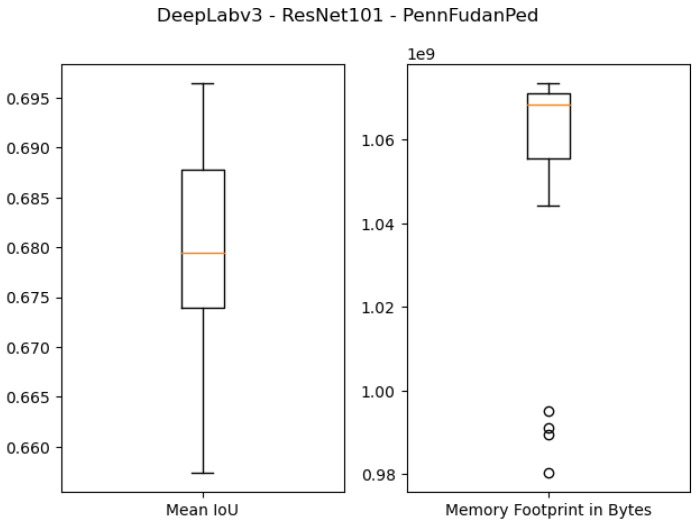
Boxplot results for the *Penn–Fudan* dataset using the *ResNet101* backbone and 1 GB of desired memory footprint.

The quantitative results of using 1 GB as the specified memory footprint of the pruned solutions in our algorithm are presented in the first four rows of Table 3 and Figure 7, Figure 8, Figure 9 and Figure 10. For the ResNet50 Backbone, the best test Intersection-over-Union (IoU) values obtained were equal to 0.9539 and 0.7199 for the São Carlos Rivers and Penn–Fudan datasets, respectively. Additionally, these two candidate solutions had a memory footprint of $1.01\times 10^{9}$ and $1.02\times 10^{9}$ bytes, respectively. In the case of the ResNet101 backbone, the best test IoU values were equal to 0.9505 and 0.6964 for the São Carlos Rivers and Penn–Fudan datasets, respectively. Additionally, these candidate solutions had a memory footprint of $1.07\times 10^{9}$ bytes.

**Figure 11:**
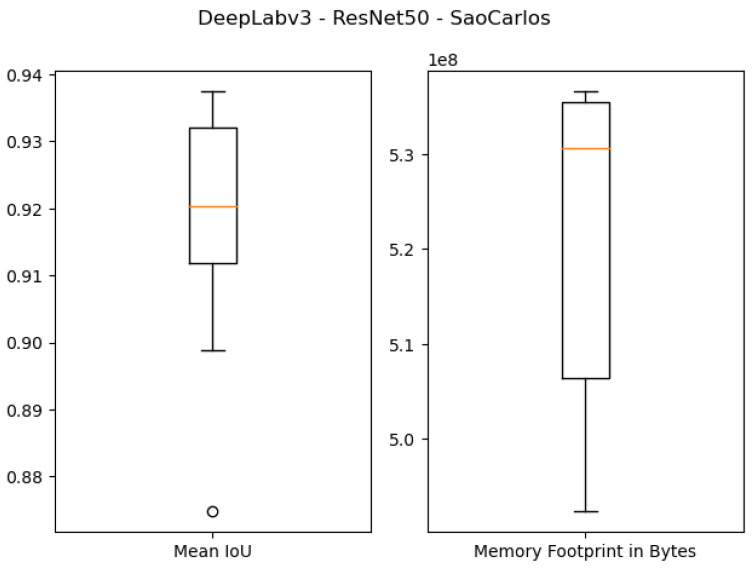
Boxplot results for the *São Carlos Rivers* dataset using the *ResNet50* Bhckbone and 512 MB of desired memory footprint.

**Figure 12:**
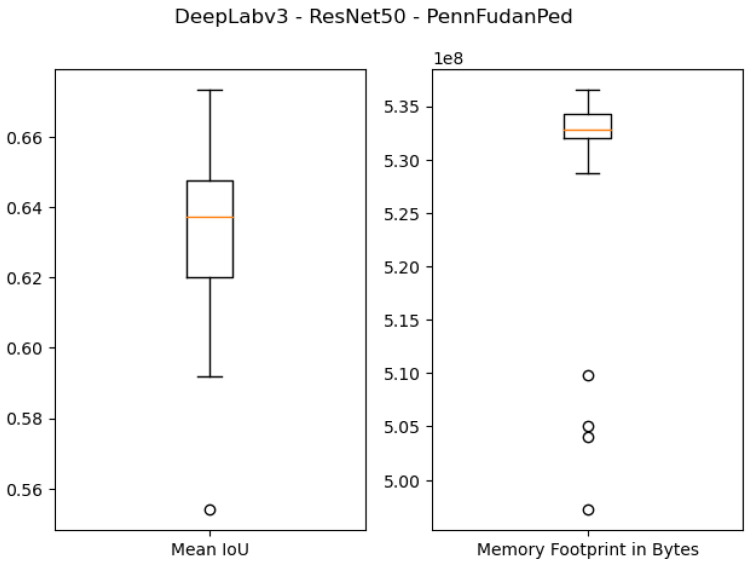
Boxplot results for the *Penn–Fudan* dataset using the *ResNet50* backbone and 512 MB of desired memory footprint.

**Figure 13:**
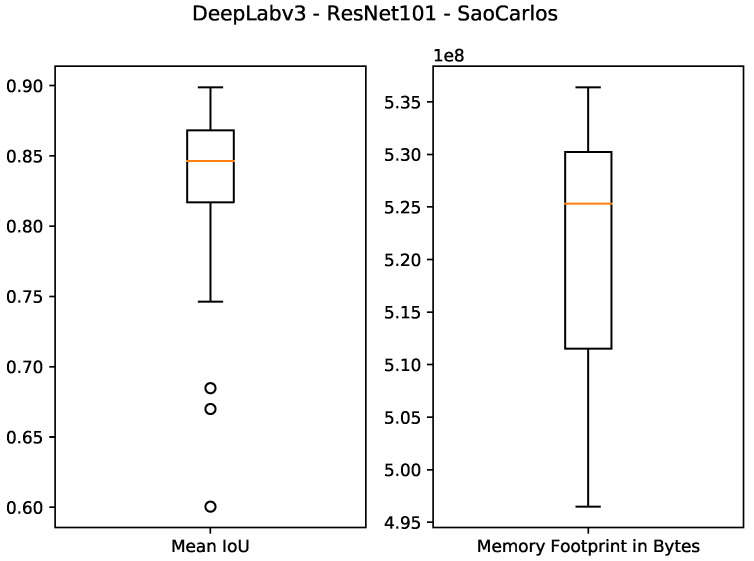
Boxplot results for the *São Carlos Rivers* dataset using the *ResNet101* backbone and 512 MB of desired memory footprint.

**Figure 14:**
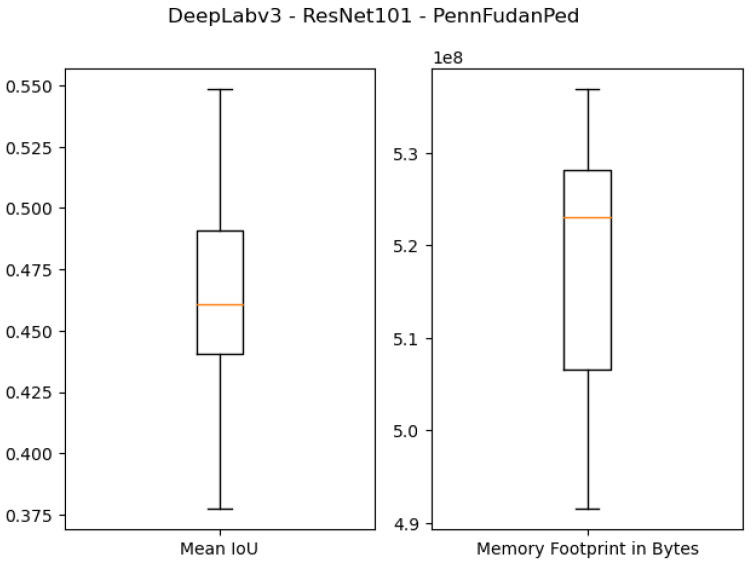
Boxplot results for the *Penn–Fudan* dataset using the *ResNet101* backbone and 512 MB of desired memory footprint.

The results of using 512 MB as the desired memory footprint are presented in the last four rows of Table 3 and Figure 11, Figure 12, Figure 13 and Figure 14. For DeepLabv3 using ResNet50 as a backbone, the test IoU values were equal to 0.9374 and 0.6734 for the *São Carlos Rivers* and *Penn–Fudan* datasets, respectively. With ResNet101 as a backbone, the IoU values on the test sets were equal to 0.8988 and 0.5482 on the *São Carlos Rivers* and *Penn–Fudan* datasets. Concerning the memory footprint of the best candidate solutions, the models using the ResNet50 backbone were pruned to a memory footprint of $5.06\times 10^{8}$ and $5.34\times 10^{8}$ bytes on both datasets. Likewise, the models using the ResNet101 backbone had a memory footprint of $5.31\times 10^{8}$ and $5.29\times 10^{8}$ bytes, respectively.

### 5.2. Discussion

From the results presented previously, we can see that the proposed pruning strategy can successfully find DNN models with the desired memory footprint. Furthermore, from 30 independent runs, our algorithm’s candidate solutions had memory footprints below the specified footprint size.

**Figure 15:**
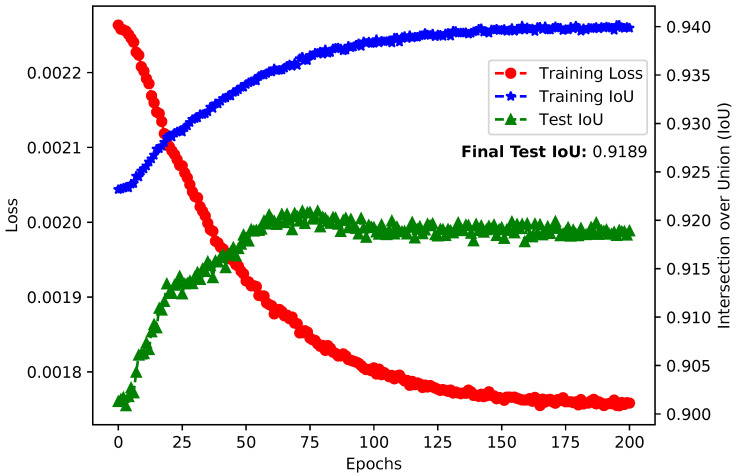
Retraining results of the best ResNet101 model with 512 MB of memory footprint on the *São Carlos Rivers* dataset.

Concerning the performance of the pruned DNN solutions, from Table 3, we can see that the pruned models with 1 GB of memory footprint perform better regarding segmentation than the models with 512 MB of memory footprint. We can observe that this comes from the fact that a memory footprint of 1 GB is equal to a decrease of 4.46% and 44.85% for the ResNet50 and ResNet101 backbones, while 512 MB corresponds to a 52.05% and 72.32% decrease, respectively, meaning that with 1 GB of memory footprint fewer convolutional filters are eliminated than with 512 MB. However, this result also comes from the fact that we are using the same fine-tuning parameters for all candidate solutions from both datasets. To prove this statement, we retrained the best ResNet101 model with a 512 MB memory footprint on the São Carlos Rivers dataset to verify if we could recover some of its segmentation performance. This new training session was performed for 200 epochs, an initial learning rate of $10^{-6}$, and a learning rate decay schedule of 20 epochs. As a result of this retraining session, illustrated in Figure 15, the model reached a test IoU equal to 0.9189 from the previous 0.8988, which is significantly closer to the original model’s performance of 0.9404, but with a reduction in memory footprint of 72.63%.

**Figure 16:**
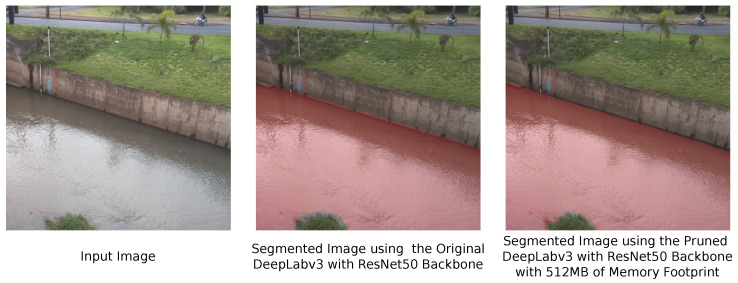
Semantic segmentation results of the pruned DeepLabv3 with ResNet50 backbone model with 512 MB of memory footprint.

Additionally, we tested the segmentation performance of the DeepLabv3 with ResNet50 backbone pruned to have a memory footprint of 512 MB (the model in the fifth row on Table 3), a reduction of 53% compared with the original model, with an image of the river we are monitoring in São Carlos city. Figure 16 presents the segmentation results for this model. We can see that, even though we eliminated 53% of the memory footprint of this DNN model, it can still perform a highly accurate segmentation compared with the original model. The segmentation in Figure 15 shows that our proposed DNN architecture pruning algorithm can reduce the memory footprint of DNN models while maintaining their performance.

Lastly, we also compared the proposed algorithm with two peer competitors ones. In this case, to make our results comparable with those from our peers, we used our algorithm to reduce the model’s number of floating-point operations (FLOPs) instead of the memory footprint. Specifically, we compared our results with those reported by Li et al. in [16] and Ding et al. in [34] on the CIFAR10 dataset for a multi-label classification task, using the VGG16, ResNet56, and ResNet110 DNN models. We also target the highest pruning rate reached by these peer competitors’ algorithms. For the VGG16 model, we target a decrease of 81.39% on the number of FLOPs. Likewise, for the ResNet56 and the ResNet110, we target a decrease of 66.88% and 38.6%, respectively. Table 4 presents our pruning results compared with those from Li et al. [16] and Ding et al. [34], and it shows that our algorithm was capable of reducing the number of FLOPs to the specified amount while maintaining a competitive classification performance, with a difference of less than 1% on the test set. These results show that our randomized pruning strategy is competitive with those based on complex selection criteria.

### 5.3. Deployment Guidelines

If one would like to deploy the proposed river flooding detection system and the memory-based DNN pruning algorithm on real hardware, it is essential to follow these guidelines:

1. The river flooding detection system requires some flat surface orthogonal to the river’s water blade for use as a reference;
2. It is necessary to determine how much memory the IoT or edge device can spare to run a DNN model. In our case, because our sensor has a total memory of 1 GB, a DNN with 512 MB of memory footprint is ideal, keeping the other 512 MB of memory to other tasks;
3. The proposed memory-based pruning algorithm must run in a powerful workstation equipped with a powerful GPU;
4. Once a suitable pruned DNN has been found and trained in the problem at hand, this model can be directly deployed in the IoT or Edge device to perform its detection tasks.

Furthermore, it is important to note that the proposed algorithm uses the supervised training paradigm. Thus, the pruning procedure must be repeated if one wants to use new data to retrain the DNN model. However, once a suitable pruned DNN model is found, it can be deployed at the edge device without the need to use a powerful workstation.

## 6. Conclusions and Future Work

In order to manage flood risk, different solutions may be deployed in an environment which may require that flooding must be detected automatically. A type of data that can be made readily available are live images from cameras placed at the basins of a river. Since this type of application is critical in the sense that a false negative may prevent a damage control solution from being triggered, it is undesirable for the flood detector to depend on volatile infrastructure components, such as mobile internet connection. One solution to tackle this issue is deploying the detection mechanism in the edge (i.e., within the IoT device), which may be challenging when dealing with the resource-greedy neural networks usually employed for computer vision tasks.

In the present work, we propose a DNN architecture pruning algorithm intended to reduce a given DNN model’s memory footprint. Our pruning strategy consists of the elimination of convolutional filters of each convolutional layer. Different from others’ work, our algorithm does not require the use of any filter metric, such as the average percentage of zeros or the entropy. The proposed algorithm also does not rely on the use of an evolutionary framework. It can find viable pruned solutions by randomly eliminating convolutional filters and choosing a candidate solution with the highest segmentation performance.

Our proposed pruning algorithm was tested with two semantic segmentation datasets. One of these datasets is from our wireless sensor network (WSN), which monitors urban river flooding events in São Carlos, SP, Brazil. The algorithm was set up to prune DNN models to have two memory footprints: 1 GB and 512 MB. We consider these values to be adequate to deploy DNN models on memory-constraint devices, such as those used in our WSN. In that sense, memory-constraint devices, such as those used in Internet of Things (IoT) applications, can take advantage of the excellent learning capabilities of DNN models. Furthermore, our results show that the pruned models still have a competitive segmentation performance compared to the original models. In some cases, the pruned models’ performance surpassed the original models, showing that our proposed algorithm is adequate to prune DNN models to be applied to IoT applications.

For future works, our first step is to upgrade our sensor network to employ the pruned DNN models on each node, allowing the flooding detection to work both through the internet and locally in case of problems with the sensors‘ connection. Then, we will apply the proposed pruning algorithm with DNN models used for different tasks, such as object detection and classification, and use the knowledge from this work to develop an algorithm capable of partitioning a DNN model to run on multiple nodes of an IoT sensor network when pruning a model is not feasible. We are also working on our river flooding algorithm itself to make it compatible with a variety of natural streams, such as rivers without concrete flooding walls.

## Figures and Tables

**Table 1:** Memory footprint and test IoU of the original DeepLabv3 models.

DeepLabv3 Backbone	Dataset	Original Memory Footprint	Test IoU
ResNet50	São Carlos Rivers	$1.12\times 10^{9}$ bytes	0.9404
	Penn–Fudan		0.6992
ResNet101	São Carlos Rivers	$1.94\times 10^{9}$ bytes	0.9412
	Penn–Fudan		0.6779

**Table 2:** Parameters used to evaluate the proposed algorithm.

**Pruning Parameters**	
Number of candidate solutions (*N*)	30
Desired memory footprint (mem) in bytes	$1.07\times 10^{9}$ and $5.37\times 10^{8}$
Probability of removing a filter (*p*)	0.1
Learning rate for memory evaluation ($\alpha_{mem}$)	$10^{-4}$
**Training Parameters**	
Number of epochs for fine-tuning (*e*)	100
Learning rate for fine-tuning (α)	$10^{-5}$
Learning rate decay schedule step (*s*)	7
Learning rate decay (γ)	0.5

**Table 3:** Best Results obtained with the proposed pruning algorithm.

DeepLabv3 Backbone	Dataset	Best Test IoU	Memory Footprint (in Bytes)	Percentual Decrease in Memory Footprint
**Desired Memory Footprint:**$1.07\times 10^{9}$ bytes or 1 GB				
ResNet50	São Carlos Rivers	0.9539	$1.01\times 10^{9}$	9.82%
	Penn–Fudan	0.7199	$1.02\times 10^{9}$	8.93%
ResNet101	São Carlos Rivers	0.9505	$1.07\times 10^{9}$	44.85%
	Penn–Fudan	0.6964	$1.07\times 10^{9}$	44.85%
**Desired Memory Footprint:**$5.37\times 10^{8}$ bytes or 512 MB				
ResNet50	São Carlos Rivers	0.9374	$5.06\times 10^{8}$	54.82%
	Penn–Fudan	0.6734	$5.34\times 10^{8}$	52.32%
ResNet101	São Carlos Rivers	0.8988	$5.31\times 10^{8}$	72.63%
	Penn–Fudan	0.5482	$5.29\times 10^{8}$	72.73%

**Table 4:** Pruning results from peer competitors using the CIFAR10 dataset.

Approach	DNN Model	% FLOPs Pruned	Test Error
Li et al. [16]	VGG16	34.2%	6.60%
	ResNet56	27.6%	6.94%
	ResNet110	38.6%	6.70%
Ding et al. [34]	VGG16	81.39%	7.56%
	ResNet56	66.88%	9.43%
**Ours**	**VGG16**	**81.69%**	**8.28%**
	**ResNet56**	**68.79%**	**10.43%**
	**ResNet110**	**39.91%**	**7.13%**

## Data Availability

The data presented in this study are openly available in GitHub at https://github.com/feferna/UserBasedMemoryPruningDNN (accessed on 20 September 2021).
